# P04.21. What infectious disease (ID) physicians believe about integrative medicine (IM) modalities

**DOI:** 10.1186/1472-6882-12-S1-P291

**Published:** 2012-06-12

**Authors:** K Shere-Wolfe, J Tilburt, C D'Adamo, B Berman, M Chesney

**Affiliations:** 1 University of Maryland, Baltimore, USA; 2 Mayo Clinic, Rochester, USA; 3 University of California, San Franscisco, USA

## Purpose

The purpose of this study was to assess ID physicians’ beliefs regarding the usefulness of IM modalities and how they work.

## Methods

A national survey of 1000 practicing ID physicians in the United States was conducted in 2010. We asked ID physicians to rate their degree of agreement with statements about IM modalities (“strongly agree”, “somewhat agree”, “somewhat disagree”, and “strongly disagree”). The statements were regarding IM modalities’ (1) usefulness, (2) placebo mechanism, (3) utility in alleviating symptoms, (4) effect on the underlying disease process, and (5) ability to directly affect the immune system. We then asked a subset of the respondents who indicated that they either “strongly agree” or “somewhat agree” to the statements “In general, IM modalities affect the underlying disease process” or “In general IM modalities, affect the immune system” to indicate which areas of IM they were referring.

## Results

A total of 311 (31%) responded to the survey. Unless indicated otherwise, 297 participants responded to these set of questions. Seventy-five percent of respondents (n=301) agreed (strongly or somewhat) that IM modalities were useful. Approximately half of the respondents (51%) believed that IM modalities derive their benefit from the placebo effect. Seventy-two percent agreed that IM modalities alleviate symptoms. About half the respondents agreed that IM modalities affect the disease process and the immune system (53% and 53%, respectively). This last subset of respondents (n=157) believed the following IM categories could affect the disease process and or immune function: (1) Mind body medicine (n=90), (2) Botanicals/Supplements (n=96), (3) Manipulative and body based modalities (n=63), (4) Energy medicine (n=24), and (5) Whole medical systems (n=35).

## Conclusion

The majority of respondents felt that IM modalities are useful in patient care.

